# Supplementary material for: Noncompetitive Chromogenic Lateral-Flow Immunoassay for Simultaneous Detection of Microcystins and Nodularin
Source: Biosensors (Basel). 2019 Jun 18;9(2):79. doi: 10.3390/bios9020079 (PMC6627203; doi:10.3390/bios9020079)
Supplement: Supplementary file 1 [file biosensors-09-00079-s001.pdf]

Supplementary Materials:

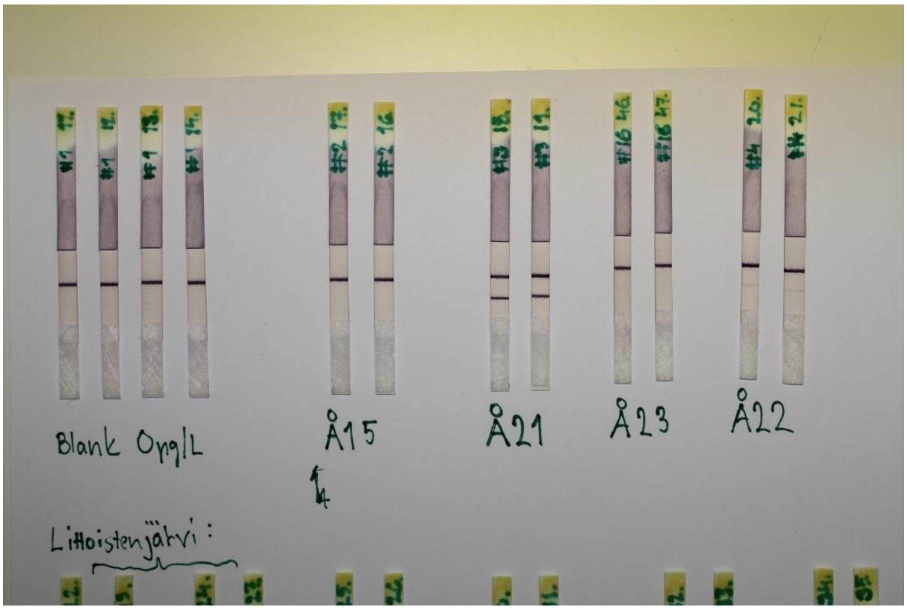

Figure S1. LFIA with blank and environmental samples set1.

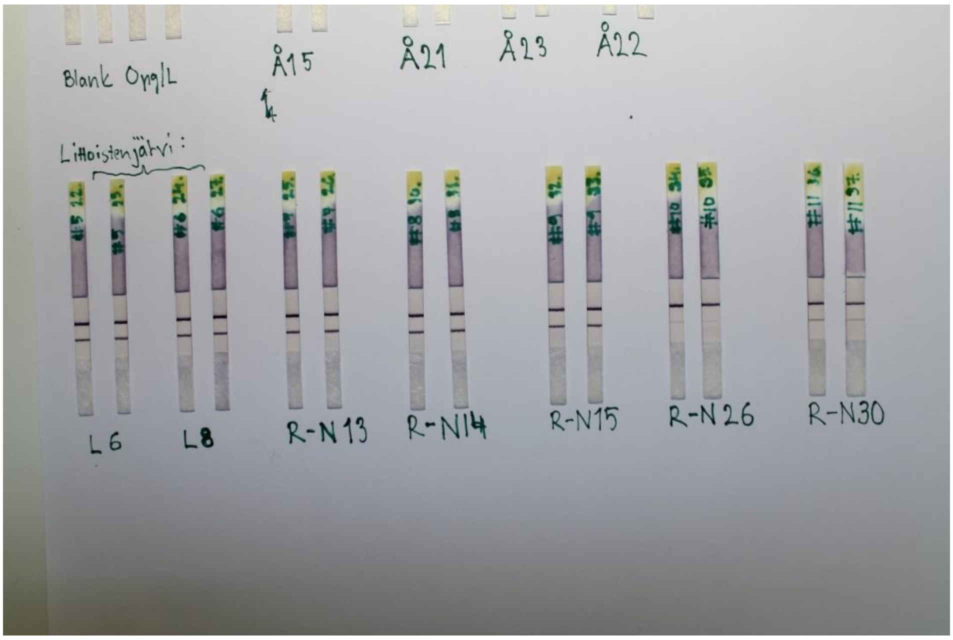

Figure S2. LFIA with environmental samples set2.

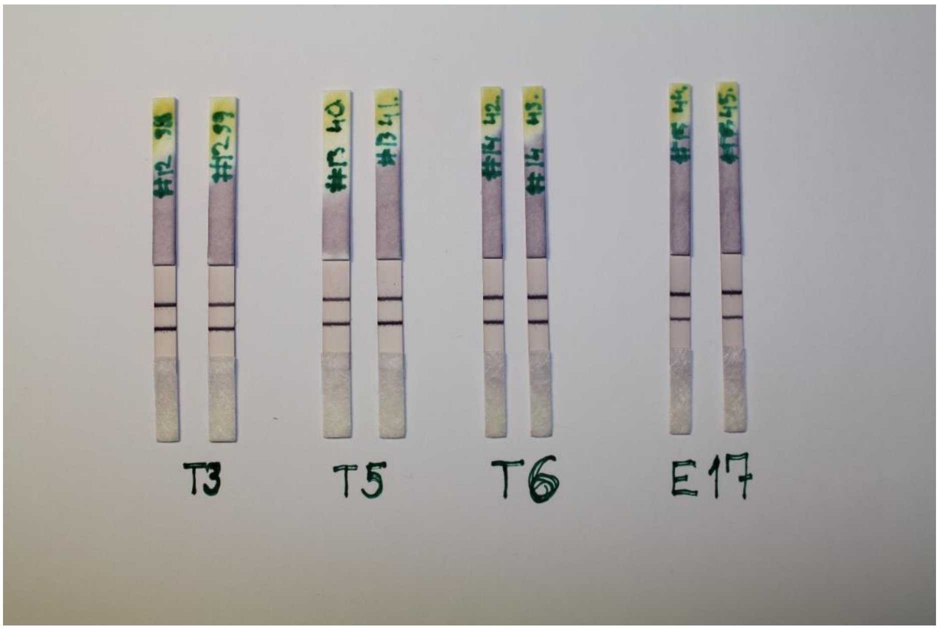

Figure S3. LFIA with environmental samples set3.
